# Good results with cemented total hip arthroplasty in patients between 40 and 50 years of age: 168 hips followed for 2–19 years

**DOI:** 10.3109/17453671003717831

**Published:** 2010-04-06

**Authors:** Daniël C J de Kam, Jean W M Gardeniers, René P H Veth, B Willem Schreurs

**Affiliations:** Department of Orthopaedic Surgery at Radboud University Nijmegen Medical Centre, Nijmegenthe Netherlands

## Abstract

**Background and purpose** Total hip arthroplasties in young patients have lower long-term survival rates than in older patients. We evaluated the use of a unique treatment protocol in patients aged between 40 and 50 years. In all cases we used a cemented THA, and for acetabular deficiencies we also used impacted bone grafts together with a cemented cup.

**Methods** In 140 consecutive patients who were between 40 and 50 years of age at index surgery, 168 cemented total hip prostheses were evaluated after a mean follow-up time of 10 (2–19) years. Acetabular deficiencies were reconstructed with wire meshes and impacted bone grafts with a cemented cup (70 hips). During follow-up, 18 patients died (27 hips); in this group 3 hips (3 patients) had been revised. None of the patients were lost to follow-up. In all surviving patients, clinical assessment was performed with hip-score questions and all radiographs were evaluated.

**Results** All clinical questionnaires showed an improved clinical hip score. 29 hips (17%) were revised after a mean of 8 (0.3–18) years. Kaplan-Meier survival analysis showed a survival of 88% (95% CI: 82–94) after 10 years with revision of either component for any reason. Survival with endpoint revision for aseptic loosening of either component was 94% (95% CI: 90–99) after 10 years.

**Interpretation** Cemented implants in young patients have satisfying long-term results. Reconstruction of acetabular deficiencies with impacted bone grafts show promising results.

## Introduction

The outcome of total hip arthroplasty in patients who are less than 50 years old is less favorable than in older patients (Learmonth et al. 2007). Thus, many designs and modifications have been tried during the past 25 years to improve the outcome in younger patients, including cemented hips, uncemented hips, hybrids, and resurfacing hip arthroplasty.

We have also used cemented total hip implants with a metal-on-polyethylene bearing in younger patients. In cases of loss of acetabular bone stock, which is common in these patients, we have reconstructed the bone defects using bone impaction grafting and a cemented cup. We have consequently used this method for the last 30 years, which guarantees no selection bias using. Being a referral center, we accept all patients, we do not refer any, and we do not perform surgery on patients who are part of this cohort outside our center.

We analyzed the outcome for all patients who were between 40 and 50 years of age at the time of surgery, over a 16-year period.

## Patients and methods

### Patients

All patients aged between 40 and 50 years who underwent a primary THA at our department between January 1988 and July 2004 were included in the study. All indications were included, with the exception of oncological cases. We only used cemented THAs, and in cases with acetabular deficiencies these defects were reconstructed with bone impaction grafting together with a cemented cup. 168 consecutive hips in 140 patients (74 females) were operated (Table 1). 28 patients had a bilateral THA. The mean age at index surgery was 46 (40–49) years. 58 patients were class A, 13 were class B, 49 were class BB, and 48 were class C according to the modified Charnley classification (Roder et al. 2006).

**Table 1.** Indications for primary total hip arthroplasty

Indications	No. of hips	
Congenital hip dysplasia	39	
Rheumatoid arthritis	24	
Perthes' disease	5	
Avascular necrosis eci	6	
Epiphyseal dysplasia	2	
Posttraumatic osteoarthritis	11	
Bechterew's disease	1	
Posttraumatic avascular necrosis	5	
Synovitis villonodularis pigmentosa	1	
Epiphysiolysis	6	
Coxitis	3	
Protrusio acetabuli	7	
Osteomyelitis	2	
Primary osteoarthrosis	17	
TBC coxitis	3	
Achondroplasia	1	
Osteopetrosis	1	
Alcohol-induced avascular necrosis	1	
Corticosteroid-induced avascular necrosis	33	
Kidney transplantation		14
Hodgkin's disease		6
Aplastic anemia		3
Non-Hodgkin lymphoma		2
Myelodysplastic syndrome		2
Henoch Schonlein disease		1
Eczema		1
Colitis ulcerosa		1
Chronic obstructive pulmonary disease		1
Pulmonary sarcoidosis		1
Vasculitis		1
Total	168	

During follow-up, 18 patients died (27 hips), none of which were due to causes related to the arthroplasty. All of these patients were followed until death, and their data are included. 2 patients (2 hips) were unable to have a clinical and radiographic review; both were interviewed by telephone and stated that the hip was functioning well. The mean follow-up time for all patients was 9.7 (2.0–19.3) years. The mean follow-up time for patients who were available for review (deceased and revised patients excluded) was 10.3 (2.7–19.3) years.

### Implants

We used only cemented implants. Femoral stems inserted were: Exeter (n = 75) (Stryker Howmedica, Newbury, UK), Charnley/Charnley Elite/Charnley Elite+ (n = 22, 31, and 23, respectively) (DePuy, Leeds, UK) and the Müller Straight Stem (n = 17) (Sulzer, Wintherthür, Switzerland). The Charnley stems were the only collared stems used. Mean follow-up times were 16 years for the Müller implants, 12 years for the Charnley implants, and 6 years for the Exeter implants.

The acetabular components used were: Exeter/Contemporary cups with an inner diameter of 28 mm (n = 63) and 22 mm (n = 1) (Stryker Howmedica), Charnley/Charnley Elite/Charnley Elite+/Ogee cups with an inner diameter of 22.225 mm (n = 10) or 28 mm (n = 72) (DePuy) and Müller cups with an inner diameter of 28 mm (n = 3) and 32 mm (n = 19) (Sulzer). All cups were made of conventional high-molecular-weight polyethylene and all femoral heads were made of a cobalt-chrome alloy; no metal-backed liners or ceramic femoral heads were used.

72 hips (43%) had an acetabular deficiency. According to the AAOS classification (D'Antonio et al. 1989), 19 hips had a segmental defect (type I), 41 had a cavitair defect (type II), and 12 had a combined segmental and cavitair defect (type III).

### Surgery

All patients were given antibiotics prophylactically before surgery. All operations but one were performed using a posterolateral approach, the exception being anterolateral. No trochanteric osteotomies were performed. Of the 72 hips with acetabular deficiencies, in 70 the defect was reconstructed with bone impaction grafting and, if indicated, metal meshes (Schreurs et al. 2001). In 2 hips with segmental rim defects, the defect was reconstructed with a solid graft. In 2 cases only allograft was used because the femoral head was not suitable for grafting; in 67 cases autograft was used; in 3 cases both allograft and autograft were used because of an extensive acetabular defect.

Both prosthetic components were inserted with second- or third-generation cementing techniques with vacuum-mixed antibiotic-loaded cement, cement pressurizing with a cement gun, pulse lavage, and a distal intramedullary femoral plug. Patients with large acetabular reconstructions were mobilized with partial weight bearing, which was increased to full weight bearing after 12 weeks. Cases of extensive acetabular reconstruction had a bed-rest period of up to 6 weeks. Postoperatively, patients received oral anticoagulants for 3 months or low-molecular-weight heparin for 6 weeks. NSAIDs were given to prevent heterotopic ossification.

### Clinical evaluation

All patients were followed periodically at our outpatient clinic with an interview, a physical examination, and radiographs. Clinical outcome was evaluated with several questionnaires: the Harris hip score (HHS) (Harris 1969), the Oxford hip questionnaire score (OHQS, since 1998) (Dawson et al. 1996) and visual analog scales (VAS, 0–100) assessing pain at rest, pain during physical activities (worst score 100), and satisfaction (best score 100). Clinical questionnaires were obtained by independent researchers at our outpatient clinic.

### Radiographic evaluation

The pre- and postoperative anteroposterior and lateral radiographs of every patient were assessed for position (Salvati et al. 1976), incorporation of grafts, acetabular defects (D'Antonio et al. 1989), position of wire meshes, heterotopic ossifications (Brooker et al. 1973), polyethylene wear (Dorr and Wan 1996), rounding-off of the calcar, and radiolucent lines. Loosening of the cup was defined as a demarcation in ≥ 2 zones around the acetabular component of ≥ 2 mm, progressive demarcation, component migration of ≥ 3 mm, component tilting of ≥ 8°, and/or cement or prosthesis fracture. We recorded the acetabular radiographic changes according to the zones of DeLee and Charnley (1976). Determination of cup migration was measured in relation to the inter-teardrop line instead of the Kohler line (Goodman et al. 1988). Definite loosening of the stem was defined according the criteria of Harris et al. (1982). Femoral radiographic changes were recorded using the Gruen zones. All measurements were corrected for radiographic magnification.

### Statistics

Kaplan-Meier cumulative survival analysis was used to calculate survival rates. Endpoints used for survival analysis were: revision for any reason, revision for any reason excluding infections, revision for aseptic loosening, and radiographic loosening. Survival rates were calculated for the whole THA and for the cup and stem separately. The survival rates at 10 and 12.5 years are reported, because until 12.5 years more than 25% of the patients still remained in the study population. The Student t-test and chi-squared test were also used. For finding differences in survival, the log-rank test was used. A p-value of < 0.05 was considered significant.

## Results

### Clinical results

All postoperative scores for the clinical questionnaires improved. The median HHS improved from 51 (2–79) points (n = 110) to 94 (10–100) points (n = 126) (p < 0.001). The median OHQs (best score is 12 and the worst score is 60) improved from 38 (12–57) points (n = 24) to 16 (12–45) points (n = 121) (p < 0.001). The median postoperative VAS scores for pain at rest, pain during physical activity, and satisfaction were 0 (0–80) points (n =126), 0 (0–90) points (n = 126), and 100 (0–100) points (n = 124), respectively.

### Revisions

**Figure 1.**
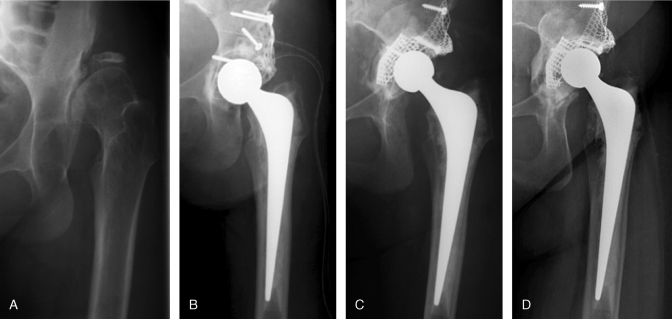
A 43-year-old female with developmental dysplasia of the hips with high dislocation. A. Preoperatively. B. Directly postoperatively after primary THA with distalization and reconstruction of the cup to its anatomical center of rotation giving a neurological deficit. C. Postoperatively after cup revision 2 years later with cranialization of the cup. D. 12 years after cup revision.

After a mean follow-up of 9.7 (2–19.3) years, 29 hips (17%) were revised (Table 2). Mean time to revision was 8.1 (0.3–18.4) years. The main reasons for revision were septic loosening (n = 5), recurrent dislocation (n = 5), and aseptic loosening (n = 15). No statistically significant differences were found between the cups with and without impacted bone grafts in terms of time to revision and the number of revisions. Remarkably, all the hips revised for septic loosening and all the cups revised because of recurrent dislocations were THAs without an acetabular reconstruction. Of the hips revised because of infection, only 1 patient had had an infection within 2 years (at 0.9 years). All other revisions were performed because of late infections. 2 stems fractured: in 1 case an Exeter stem and in the other a Charnley Elite stem. In one patient with a high dislocation of the hip because of developmental dysplasia (Figure 1A), an acetabular reconstruction was performed at the original center of rotation and the cup was distalized (Figure 1B). As a result of this distalization, tension on the sciatic nerve resulted in a persistent sensory and motor deficit. After 2.4 years, the cup was revised to a higher position (Figure 1C) and the neurological deficit partly recovered. The percentages of revisions were spread equally over time for both implant types and diagnoses.

**Table 2.** Reasons for revision and components revised

Reason for revision	THA	Cup	Stem	Total	Mean time (yr) to revision (range)
Aseptic loosening	9	5	1	15	10 (2.5–18)
Septic loosening	5	0	0	5	5.0 (0.9–13)
Recurrent dislocations	0	4	1	5	4.1 (0.3–11)
Fracture	0	0	2	2	8.5 (6.6–10)
Traumatic loosening	0	1	0	1	15
Neuropathy	0	1	0	1	2.4
Total	14	11	4	29	8.1 (0.3–18)

### Radiographic results

For 2 patients, the most recent radiograph was missing. 23 hips were radiographically loose. In 16 of these cases the cup, in 6 cases the stem, and in 1 case both stem and cup were loose. 18 were revised.

The average wear in the non-revised hips was 0.11 (0.00–1.23) mm/year and the average wear in the revised hips was 0.26 (0.00–1.04) mm/year (p < 0.001). Excessive wear of > 0.1 mm/year was observed in 77 hips (23 revised and 54 unrevised) (p < 0.001). The wear for cups reconstructed with impacted bone grafts and for cups implanted without bone grafts was similar. An abnormal position of the cup was not associated with higher wear. Radiographically loose cups showed higher wear rates than radiographically stable cups (mean 0.23 vs. 0.13 mm/year) (p = 0.01).

All grafts became incorporated and none showed signs of osteolysis. However, in some cases the meshes used blurred the examination of incorporation of the grafts. Cups reconstructed with impacted bone grafts showed less radiolucent lines and less acetabular osteolysis. 51 hips had heterotopic ossification: 25 of Brooker class I, 16 of class II, and 10 of class III.

### Complications

In addition to the 5 revisions done for recurrent dislocation, 14 other patients (8%) had 1 or more hip dislocations; all of these were treated nonoperatively. 2 patients had pain and limited hip motion, and had heterotopic ossifications excised. 4 patients had temporary nerve palsies (3 cases with developmental dysplasia of the hips and 1 case after septic coxitis in childhood). A postoperative superficial wound infection occurred in 4 patients and healed with antibiotics. In 1 case, a postoperative hematoma was evacuated because of pressure on the sciatic nerve. Cement that had leaked through DHS screw holes at the greater trochanter was removed because of pain in 1 patient.

### Survival analysis

**Figure 2.**
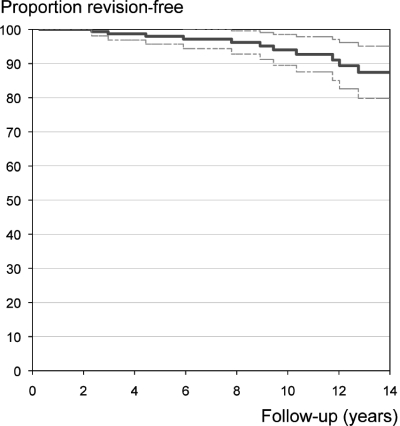
Kaplan-Meier survival curve with endpoint revision for aseptic loosening of any component (95% confidence intervals in broken lines).

Kaplan-Meier survival analysis with revision of any component for any reason as endpoint gave survival rates of 88% at 10 years and 80% at 12.5 years (Table 3). When infections were excluded, the survival rates increased to 91% at 10 years and 83% at 12.5 years. Survival with revision of any component for aseptic loosening as endpoint was 94% at 10 years and 89% at 12.5 years (Figure 2). There were no statistically significant differences in survival between the different Charnley classifications. With the endpoints revision for any reason and revision for aseptic loosening, the survival rates were similar in patients with bilateral or unilateral THA (p = 0.3 and p = 0.5); thus, no outcome bias concerning bilateral or unilateral THA was present (Bryant et al. 2006).

**Table 3.** Survival rates (95% CI) with all endpoints at 10 and 12.5 years

Component	Any reason		Any reason excl. infections		Aseptic loosening	
	10 years	12.5 years	10 years	12.5 years	10 years	12.5 years
THA	88 (82–94)	80 (72–88)	91 (85–96)	83 (75–91)	94 (89–99)	89 (83–96)
Stem	92 (87–97)	88 (81–95)	95 (91–99)	91 (84–97)	97 (93–100)	94 (88–99)
Cup	90 (84–95)	84 (77–91)	92 (88–97)	86 (79–93)	94 (89–99)	91 (84–97)

## Discussion

We believe that our findings are relevant and reliable because this was a large series of consecutive total hip implantations with no patients lost during follow-up (Murray et al. 1997). The approach used was the only one, so no selection bias was created. Our study is not a single-surgeon study, however; the operations were performed by several surgeons.

One limitation of our study was the short follow-up in some patients. Another limitation is that we used different kinds of implants over time; however, all were cemented with a full polyethylene cup. The Exeter stems had shorter follow-up; thus, the long-term follow-up mostly depended on the results of the Charnley and Müller implants. Nowadays, we only use the Exeter stem and Exeter/Contemporary cups routinely in primary THA, and also Müller (32-mm) cups in revision arthroplasties. Because of the wide range of sizes and offsets of the Exeter stem, no custom-made implants are necessary.

Several studies have shown that an acetabular reconstruction with bone impaction grafting creates a unique bone-cement-cup interface (Heekin et al. 1995, van der Donk et al. 2002). This could be an explanation for the smaller amount of radiolucent lines we found in the cups reconstructed with bone impaction grafting, as also reported by De Kam et al. (2009).

Notably, the outcome in our patients who had a cemented THA and cups reconstructed with bone impaction grafting are similar to the results of Charnley THA in older patients (Garellick et al. 1994, Kerboull et al. 2004, Buckwalter et al. 2006, Hulleberg et al. 2008).

Good results of cemented THA, all with the original Charnley prosthesis, in patients under the age of 40 with a mean follow-up time of longer than 10 years vary between 85% and 93% survival (Joshi et al. 1993, Sochart and Porter 1997, Garcia-Cimbrelo et al. 2000, Chiu et al. 2001). In another study, the survival of cemented Charnley-Kerboull THA in patients under and over the age of 40 was the same (90% vs. 89% at 20 years) (Kerboull et al. 2004).

We have not found any reports on uncemented implants in patients under the age of 40 with a mean follow-up longer than 10 years and with a survival rate of more than 90% at 10 years, with revision for any reason of any component as endpoint. If the age limit is increased to include patients under 50, only 1 study has reported a survival rate of > 90% after 10 years of custom-made uncemented implants in 33 patients (McCullough et al. 2006). McAuley et al. (2004) reported 85% survival at 10 years with revision of any component for any reason as endpoint, in patients under 40 years of age after a mean follow-up time of 7 years. In a study on uncemented THA in patients < 55 years old in the Finnish Arthroplasty Register, the survival of different kinds of uncemented THAs at 10 years varied between 62% and 86% (Eskelinen et al. 2006).

In a meta-analysis of published literature comparing cemented and uncemented THA (Morshed et al. 2007), no advantage was found in either procedure when failure was defined as revision of any component or revision of one specific component. These authors did find a superior survival with cemented fixation in studies that included patients of all ages as compared to studies that only included patients who were 55 years of age or younger.

Higher wear rates and higher revision rates in young patients have been partly attributed to higher activity levels (Zahiri et al. 1998, Schmalzried and Huk 2004). We found similar survival rates in the different Charnley categories. However, we did not assess activity levels with proven methods such as questionnaires or accelerometers.

Despite the fact that our clinic is a training university hospital and that these institutions are known to have higher revision rates (Espehaug et al. 1999), our results are similar to those obtained with the Charnley cemented THA in younger patients. Our results highlight the value of cemented THA in this age-specific patient population. Reconstruction of acetabular defects with bone impaction grafting appears to be a promising technique.
